# *Lactobacillus delbrueckii* subsp. *bulgaricus* KLDS 1.0207 Exerts Antimicrobial and Cytotoxic Effects *in vitro* and Improves Blood Biochemical Parameters *in vivo* Against Notable Foodborne Pathogens

**DOI:** 10.3389/fmicb.2020.583070

**Published:** 2020-09-24

**Authors:** Smith Etareri Evivie, Amro Abdelazez, Bailiang Li, Shijia Lu, Fei Liu, Guicheng Huo

**Affiliations:** ^1^Key Laboratory of Dairy Science, Ministry of Education, College of Food Science, Northeast Agricultural University, Harbin, China; ^2^Department of Food Science and Human Nutrition, Faculty of Agriculture, University of Benin, Benin City, Nigeria; ^3^Department of Animal Science, Faculty of Agriculture, University of Benin, Benin City, Nigeria; ^4^Institute of Microbe and Host Health, Linyi University, Linyi, China; ^5^Department of Dairy Microbiology, Animal Production Research Institute, Agricultural Research Centre, Giza, Egypt

**Keywords:** *L. bulgaricus*, probiotics, anti-inflammation, tolerance, weight, biochemical, HDL, LDL

## Abstract

Globally, foodborne diseases (FBDs) result in millions of sicknesses and deaths annually. Cumulative evidence suggests that the use of probiotic lactic acid bacteria (LAB) strains could be a viable alternative in inhibiting the activities of foodborne pathogens. This study aims to evaluate the *in vitro* antimicrobial, cytotoxic, and tolerance levels of *Lactobacillus bulgaricus* KLDS 1.0207 against two notable foodborne pathogens – *Escherichia coli* ATCC25922 and *Staphylococcus aureus* ATCC25923. Afterward, a 48 BALB/c mice-trial was used to assess its ameliorative effects on weight and serum biochemical parameters. Results showed that the cell-free supernatant (CFS) of this strain significantly inhibited both pathogens, but these effects were abolished at pH 6.5 and 7.0 (*P* < 0.05). Also, 6.96 ± 0.02 log CFU mL^–1^ of *L. bulgaricus* KLDS 1.0207 was still viable after three hours in simulated gastric juice and at pH 3.0, indicating that this strain was a potential probiotic candidate. Also, inflammatory activities in RAW264.7 cells were significantly inhibited using 10^9^ CFU mL^–1^ of *L. bulgaricus* KLDS 1.0207 cells (*P* < 0.05). Significant weight losses were also prevented in the T_*LBSA*_ (from 19.42 ± 1.04 to 19.55 ± 0.55 g) and T_*LBEC*_ (from 22.86 ± 0.90 to 14.77 ± 9.86 g) groups compared to their respective model groups (T_*SA*_ – from 21.65 ± 1.80 to 20.14 ± 1.84, and T_*EC*_ – from 21.45 ± 0.82 to 14.45 ± 9.70 g). Besides, there was a slight weight gain in the *S. aureus* prevention group (T_*LBSA*_) compared to the model group (T_*SA*_). Serum biochemical analyses revealed that the total cholesterol (TC), triglycerides (TG), low-density lipoprotein (LDL), and some mineral levels were markedly increased by *S. aureus* and *E. coli* administrations but were reversed to normalcy in both prevention groups (T_*LBSA*_ and T_*LBEC*_). Interestingly, high-density lipoprotein (HDL) levels, which were initially disrupted in the model groups, were restored in the prevention groups (T_*LBSA*_ and T_*LBEC*_). This study presents *L. bulgaricus* KLDS 1.0207 as a promising probiotic candidate with antimicrobial, anti-inflammatory, acid, and bile tolerant and lipid-regulating applications. It also gives valuable insights for targeted future *in vivo* treatment and prevention studies involving other probiotic LAB candidates. Future *in vivo* studies elucidating specific mechanisms behind the *in vitro* antimicrobial, cytotoxic, and *in vivo* ameliorative effects are warranted.

## Introduction

Foodborne diseases (FBDs) remain a persistent cause of illnesses and mortalities worldwide. Mitigation protocols target all stages of food production from farm to fork (US Food and Drug Administration [USFDA], 2017). Of particular interest are pathogenic *Escherichia coli*, known for causing diarrhea and hemorrhagic colitis in humans (Wasey and Salen, 2019), and *Staphylococcus aureus*, reputed for its global food safety problems (Liu C. et al., 2019). Previously, the World Health Organization (WHO) had assessed the link between FBDs and the possible food sources and identifying 11 food sources that are potential reservoirs for food pathogens (Batz et al., 2005; Pires et al., 2009). This and other previous reports again emphasize the constant dangers that these pathogens can pose as they can be quickly taken in by humans and cause a variety of uncomfortable conditions (Hoffmann et al., 2017; Li et al., 2019). Besides, prompt and rapid detection of foodborne pathogens is essential to avoid FBDs (Sidari and Caridi, 2011; Kaden et al., 2018). The gut microbiota is a complex embodiment of a wide range of microorganisms that play vital roles in maintaining internal homeostasis. Tens of trillions of microbes housed here have significant effects on host metabolism, physiology, and immunity functions (Semenkovich, 2017). Several disorders, including FBDs, metabolic syndrome, cardiovascular, and kidney malfunctions, have been traced to imbalances in the gut microbiome (Evivie et al., 2017; Tang et al., 2017).

There is a growing body of *in vitro* and *in vivo* evidence that probiotics could be useful in lowering incidences of FBDs by modulating the gut microbiome (Bron et al., 2017; Evivie et al., 2017). There is increasing use of probiotic single and co-culture strains in the treatment and prevention of FBDs as they are considered a therapeutic alternative with either minimal or no known side effects (Bian et al., 2015, 2016; Evivie et al., 2017; Evivie, 2019). It thus follows that the identification and validation of probiotic LABs that could be used in the formulation of industrially important and health-promoting yogurt drinks and other food products are imperative. The Asian-Pacific region has been recently projected to becoming the biggest yogurt consumer in the world by 2023, and this presents not only immense challenges but opportunities as well (Chan et al., 2019). At present, Turkey is the second-largest yogurt consumer globally, with the United States being the lowest (Denissen et al., 2019). It has been demonstrated recently that a combination of blueberry and yogurt had therapeutic effects on obesity, type-2 diabetes, and hypertension biomarkers (Shi et al., 2019). Also, skim yogurt with microbial transglutaminase is gradually gaining awareness among yogurt consumers (García-Gómez et al., 2019). Non-fat yogurt enriched with vitamin B ameliorated homocysteine levels in obese T2D patients (Binou et al., 2019). Consumption of probiotic yogurt by healthy subjects increased the overall diversity of the oral cavity microbiome in the short-term (Dassi et al., 2018). Consumption of yogurt drink containing *Lactobacillus* strains alleviated bacteria vaginosis (BV) symptoms in female subjects (Laue et al., 2018). These products are, however, less common in many developing parts of the world or expensive even if available (Westerik et al., 2019). In addition, the strain-specificity phenomena have informed the continued research into better candidates that could enhance the industrial and health value of dairy products.

The strain *Lactobacillus delbrueckii* subsp. *bulgaricus* KLDS 1.0207, isolated from traditional dairy products from Sinkiang Province, China, has shown promising attributes recently in alleviating lead (Pb) toxicity *in vivo* in both prevention and treatment groups (Li et al., 2017). Besides, it can produce high amounts of pathogen-suppressing organic acid *in vitro*, improve immunity functions, and prevent organ damage *in vivo* (Evivie et al., 2019). However, nothing is known about its antimicrobial anti-inflammatory and tolerance properties (*in vitro*). In addition, its potential *in vivo* biochemical properties has not been reported. These findings will give further insight into its possible application as a probiotic food component and other possible therapeutic applications. The present study aims to investigate the *in vitro* antimicrobial properties of this strain against two foodborne pathogens (*Escherichia coli* ATCC25922 and *Staphylococcus aureus* ATCC25923), its cytotoxic effects in murine monocytic cell line RAW264.7 induced by lipopolysaccharides (LPS), and tolerance in simulated gastric juice and bile salts. After that, an *in vivo* study assessing its impact on weight gain and a range of biochemical parameters was performed. It is also anticipated that this study will further enhance our understanding of its potential industrial significance.

## Methodology

### Probiotics and Pathogens

The Northeast Agricultural University’s (NEAU) Key Laboratory of Dairy Science (Harbin, China) provided the *Lactobacillus bulgaricus* KLDS 1.0207 strain used in this study. Pathogenic *E. coli* ATCC25922 and *S. aureus* ATCC25923, obtained from the Heilongjiang Entry-Exit Inspection and Quarantine Bureau (Harbin, China), were prepared as described by Bian et al. (2015). For preliminary studies, the vaginal pathogen, *Gardnerella vaginalis* ATCC14018, was supplied by the Professor Xiangcheng Meng laboratory of the NEAU. All chemicals and reagents used in this research were purchased from reliable suppliers in China and of analytical grade. All experiments were carried out at the Bioengineering Unit of the KLDS, and safety precautions were strictly observed.

### Cell-Free Supernatants (CFS)

The preparation of cell-free supernatants (CFS) was performed as previously described by Bian et al. (2015) with slight modifications. Briefly, 2 mL of *L. bulgaricus* KLDS 1.0207 strain (10^8^ CFU mL^–1^) were inoculated into 100 mL of MRS broth and stored overnight at 37°C. Afterward, centrifugation was done at 10000 x g for 10 min at 4°C. The CFS obtained was neutralized, and 2 M NaOH was used to adjust to the various pH levels for this study.

### Effects of pH Alternations and Enzymatic Actions on CFS Antimicrobial Activities

The CFS of the *L. bulgaricus* KLDS 1.0207 was treated with different components - 5 mg mL^–1^, catalase, 1 mg mL^–1^, proteinase K, and 1 mg mL^–1^ papain to evaluate its antimicrobial effects against both pathogens. CFS, without any treatment, served as the control. The antimicrobial activities against *E. col*i ATCC25922 and *S. aureus* ATCC25923 after these treatments were assessed using the Oxford cup method as described previously by Bian et al. (2015), and results were presented as percentage values (%). Experiments were repeated thrice. This procedure was also followed to determine the preliminary antimicrobial activities of *L. bulgaricus* KLDS 1.0207 CFS against *G. vaginalis* ATCC14018.

### Acid and Bile Salt Tolerance Assessment

Assays testing the resistance of *L. bulgaricus* KLDS 1.0207 cells in gastric juice were carried out as earlier described by Charteris et al. (1998) with some modifications. Briefly, the strain was cultured in MRS broth at 37°C for 24 h and centrifuged at 10000 × *g* for 5 min at 4°C to collect cells. These cells were then washed twice with PBS buffer (pH 7.3) and suspended in PBS. Pepsin (0.3 mg mL^–1^) was added into PBS (pH = 1.5, 2, 2.5, and 3) to form the simulated gastric juice. Then, 3% (w/w, nearly 10^8^ CFU mL^–1^) of the washed cell suspensions were inoculated into 1 mL simulated gastric juice and 0.3 mL NaCl (0.5%, w/v), mixed and incubated at 37°C. Viable counts were calculated at 0, 1, 2, and 3 h for testing the tolerance to gastric juice during the digestion of food in the stomach. Resistance to small intestine juice and bile salts were tested in a PBS solution (pH 8.0) with 0.1 mg mL^–1^ pancreatin (Sigma) and PBS with 1% (w/v) Oxgall (Sigma), respectively, as described in Bian et al. (2015). Experiments were repeated thrice.

### Cytotoxic Effects in RAW264.7 Cells

Following the manufacturer’s instructions, the CCK-8 (Cell Counting Kit-8) method was used to assess the anti-inflammatory potentials of *L. bulgaricus* KLDS 1.0207, as recently described in Evivie et al. (2020). After digestion, RAW264.7 cells, with a density of 2 × 10^4^ mL/hole was inoculated in a 96-well plate. The supernatant was discarded, and cells were washed twice with PBS. Control and test samples were gently oscillated and incubated for 24 h. Cells were again washed twice with PBS, and then a 10 μL CCK-8 solution was added to each control and test sample. After incubating for 2 h, absorption at 450 nm was measured using an enzyme marker, and the relative survival rate of macrophages was calculated according to the following formula:

$$\mathrm{Relative}\mathrm{cell}\mathrm{survival}\left(\%\right)=\frac{\mathrm{Sample}\mathrm{absorbance}-\mathrm{Blank}\mathrm{absorbance}}{\mathrm{Control}\mathrm{absorbance}-\mathrm{Blank}\mathrm{absorbance}}\times 100$$

### Animal Husbandry and Trials

#### Animals and Experimental Design

A total of 48 BALB/c mice (7 to 8 weeks old and 20 to 25 g each) were purchased from the Vital River Laboratory Animal Technology Company (Beijing, China). Also, six metal cages with eating and drinking sections were used for the treatment groups, giving eight mice/treatment. All BALB/c mice were kept in an environment-controlled room (25°C temperature and with a 12 h light/dark cycle). The acclimatization period was for one week, during which feed and water were provided *ad libitum*. Mice in the control group were administered with 200 μL of sterile normal saline, while the other five groups were fed, as shown in Table 1. The amount of LAB strains and pathogens orally fed to the animals during the period of study was 200 and 100 μL, respectively. The CFU count of the fed LAB strain was 1 × 10^8^, and animals in each experimental group were fed twice daily. Weekly weights of the study animals were measured from acclimatization to the end of the study. This was to show the trend of the effect of the various diets on the animals. This study was approved by the NEAU Animal Ethics Committee (SRM-06).

**TABLE 1:** List of treatments administered to study animals.

S/N	Code	Content of treatment
1	C	200 μL of 0.5% saline solution
2	T_*LB*_	200 μL *Lactobacillus delbrueckii* subsp. *bulgaricus* KLDS 1.0207
3	T_*EC*_	100 μL *Escherichia coli* ATCC2522
4	T_*SA*_	100 μL *Staphylococcus aureus* ATCC2523
5	T_*LBEC*_	200 μL *Lactobacillus delbrueckii* subsp *bulgaricus* KLDS 1.0207 + 100 μL *E. coli* ATCC25922
6	T_*LBSA*_	200 μL *Lactobacillus delbrueckii* subsp. *bulgaricus* KLDS 1.0207 + 100 μL *S. aureus* ATCC25923

### Blood Biochemical Analyses

After two weeks of study, all mice were fasted and sacrificed humanely. The serum centrifuged from the blood samples was used for clinical biochemistry measurement by an automatic biochemistry analyzer (Toshiba, Tokyo, Japan). The following parameters were tested: aspartate aminotransferase (AST), alanine aminotransferase (ALT), total protein (TP), albumin (ALB), triglycerides (TG), total cholesterol (TC), high-density lipoprotein (HDL), low-density lipoprotein (LDL), urea, creatinine (CREA), sodium (Na), chlorine (Cl), calcium (Ca) and inorganic phosphorus (P).

### Statistical Analyses

Analysis of data from this research was performed by ANOVA using the SPSS v22.0 software (SPSS Inc., United States), and values were expressed as Mean values ± standard deviation (SD). LSD was used to measure significant differences between mean values at a 5% level. Mean values and SD were calculated and presented in chart form as coordinate pairs with error bars.

## Results

### Effects of Enzymatic Actions and pH Changes on CFS Antimicrobial Activities

The CFS of *L. bulgaricus* KLDS 1.0207 showed nearly 100% antimicrobial activity against both pathogens after treatment with proteinase K, papain, and a pH of 3.5 and 4.0. Catalase treatment lowered antimicrobial activities the most, compared to other enzymatic actions, giving 93.79 ± 0.45% and 90.14 ± 0.32% against *Escherichia coli* and *Staphylococcus aureus* growths, respectively (Table 2). Strain CFS antimicrobial effects reduced significantly as pH tended toward the neutral point, with antimicrobial activities against *Escherichia coli* and *Staphylococcus aureus* reducing by 86.35%, and 88.70%, respectively. No antimicrobial activities were observed at pH 6.5 and 7.0. CFS was generally more inhibitory against *E. coli* ATCC25922 than *S. aureus* ATCC25923. In this study, we also report for the first time, the potential antimicrobial effects of *L. bulgaricus* KLDS 1.0207 CFS against the vaginal pathogen, *G. vaginalis* ATCC14018 (Supplementary Table S1).

**TABLE 2:** Antimicrobial effects (%) of CFS from *L. bulgaricus* KLDS 1.0207 against *E. coli* ATCC25922 and *S. aureus* ATCC25923 after enzymatic and pH treatments.

Strain	Treatment	Control	*Escherichia coli* ATCC25922	*Staphylococcus aureus* ATCC25923
*L. bulgaricus* KLDS 1.0207	Catalase	100 ± 0^a^	93.79 ± 0.45^b^	90.14 ± 0.32^b^
	Proteinase K	100 ± 0^a^	100 ± 0^a^	100 ± 0^a^
	Papain	100 ± 0^a^	100 ± 0^a^	100 ± 0^a^
	pH 3.5	100 ± 0^a^	100 ± 0^a^	100 ± 0^a^
	pH 4.0	100 ± 0^a^	100 ± 0^a^	100 ± 0^a^
	pH 5.0	100 ± 0^a^	71.94 ± 0.28^c^	68.91 ± 0.27^c^
	pH 6.0	100 ± 0^a^	9.82 ± 0.38^d^	7.79 ± 0.39^d^
	pH 6.5	100 ± 0^a^	0 ± 0	0 ± 0
	pH 7.0	1000^a^	0 ± 0	0 ± 0

*Values were expressed as Mean ± SD. All values were determined in triplicate. Values with the same alphabet along the same row are not significantly different (P > 0.05).*

### Tolerance Assays

The acid tolerance levels of *L. bulgaricus* KLDS 1.0207 was evaluated *in vitro.* This strain showed high tolerance in simulated gastric juice after three hours of incubation and at pH 3.0, where its lowest viability loss was recorded (1.36 log CFU mL^–1^ loss) and its highest loss after three hours at pH 1.5 (7.47 log CFU mL^–1^ loss) (Table 3). Also, high viability levels were observed at pH 2, 2.5, and 3.0 for three hours, and these were significantly different from viability counts at 0 h (*P* < 0.05). Interestingly, *L. bulgaricus* KLDS 1.0207 showed high survival rates in the presence of pancreatin (pH 8.0) and bile salts (1% w/v) after three hours of incubation. Besides, *L. bulgaricus* KLDS 1.0207 showed <1 log CFU mL^–1^ loss after three hours of incubation (Table 4).

**TABLE 3:** LAB viable count under GIT conditions (log CFU mL^–1^).

pH	Time (h)	Viability count
1.5	0	8.27 ± 0.04^a^
	1	6.36 ± 0.08^b,c^
	2	3.88 ± 0.06^d^
	3	1.80 ± 0.06^e^
2.0	0	8.34 ± 0.03^a^
	1	7.16 ± 0.04^b^
	2	7.02 ± 0.03^b^
	3	6.91 ± 0.03^b^
2.5	0	8.32 ± 0.03^a^
	1	7.25 ± 0.02^a,b^
	2	7.03 ± 0.03^a^
	3	6.93 ± 0.03^b^
3.0	0	8.32 ± 0.03^a^
	1	7.27 ± 0.09^a^
	2	7.05 ± 0.04^b^
	3	6.96 ± 0.02^b^

*Values were expressed as mean ± SD. All values were determined in triplicate. Values with the same alphabet column-wise are not significantly different (P > 0.05).*

**TABLE 4:** Viable counts of *Lactobacillus bulgaricus* KLDS 1.0207 cells in intestinal juice and bile salts.

Strain	Time (h)	Pancreatin (log CFU mL^–1^)	Oxgall (log CFU mL^–1^)
*Lactobacillus bulgaricus* KLDS 1.0207	0	7.30 ± 0.06^a^	7.41 ± 0.04^a^
	1	7.27 ± 0.04^a^	7.36 ± 0.03^a^
	2	7.23 ± 0.03^a^	7.32 ± 0.02^a^
	3	7.21 ± 0.02^a^	7.30 ± 0.03^a^

*Values were expressed as mean ± SD. All values were determined in triplicate. Values with the same alphabet column-wise are not significantly different (*P* > 0.05).*

### Cytotoxic Effects of *L. bulgaricus* on RAW264.7 Cells

**FIGURE 1:**
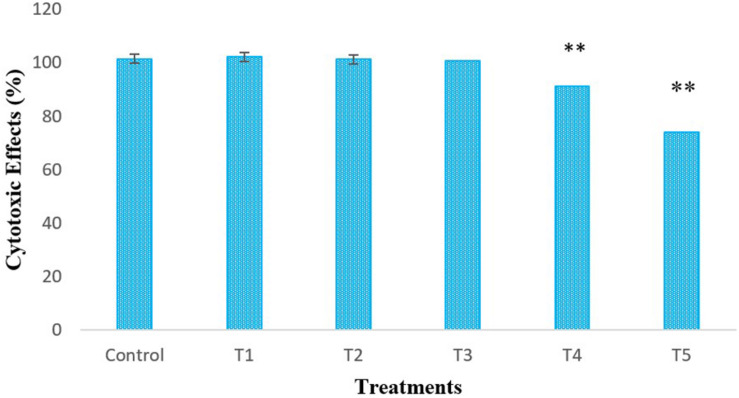
*In vitro* cytotoxic effects of different concentrations (CFU mL^–1^) of *L. bulgaricus* KLDS 1.0207 culture in RAW264.7 cells, Control - RAW264.7 cells; T1 - RAW264.7 cells + 1 × 10^5^ *L. bulgaricus* KLDS 1.0207; T2 - RAW264.7 cells + 1 × 10^6^ *L. bulgaricus* KLDS 1.0207; T3 - RAW264.7 cells + 1 × 10^7^ *L. bulgaricus* KLDS 1.0207; T4 - RAW264.7 cells + 1 × 10^8^ *L. bulgaricus* KLDS 1.0207; T5 - RAW264.7 cells + 1 × 10^9^ *L. bulgaricus* KLDS 1.0207, All values were obtained in triplicate and expressed as Mean ± SD. **Significant difference compared to the control (*P* < 0.05).

LPS-induced-RAW264.7 cells were treated with different concentrations of *L. bulgaricus* KLDS 1.0207 to determine its cytotoxic effects *in vitro* using the CCK-8 assay (Figure 1). The control was RAW264.7 cells without *L. bulgaricus* KLDS 1.0207. Results show that RAW264.7 cell activity was significantly reduced with 1 × 10^8^ and 1 × 10^9^ CFU mL^–1^ of *L. bulgaricus* KLDS 1.0207 concentrations, giving 93 and 71%, respectively (*P* < 0.05) while lower concentration levels were not significantly different from the control (*P* > 0.05).

### Weight of Study Animals

**FIGURE 2:**
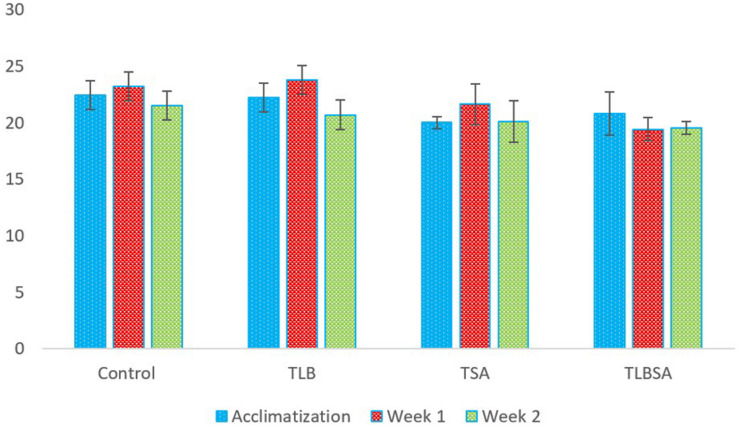
Weekly weights (g) of study animals before and after *S. aureus* ATCC25923 infection. The final weights of study animals after two weeks suggest that *L. bulgaricus* KLDS 1.0207 effectively prevented weight loss caused by *S. aureus* administration (*P* > 0.05).

**FIGURE 3:**
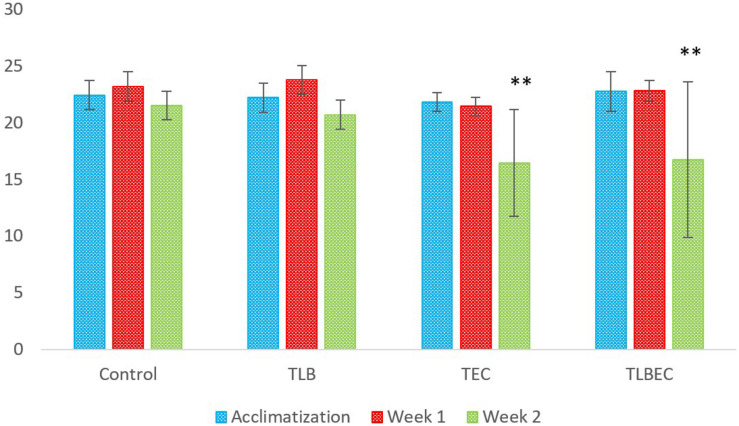
Weekly weights (g) of study animals before and after *E. coli* ATCC25922 infection. After two weeks of study, slight improvements were observed in the prevention group (T_*LBEC*_) compared to the model group (T_*EC*_) (*P* > 0.05). **Significant difference compared to the control (*P* < 0.05).

The weights (g) of mice in the six groups before, during, and after administration, with the various diets for *S. aureus*, ATCC25923 infection was evaluated (Figure 2 and Supplementary Table S2). The average weight of T_*LB*_-fed mice at the end of the first week of study was higher (23.78 ± 1.28 g) than the control (23.21 ± 1.21 g), although this was not significant (*P* > 0.05). Also, the T_*LBSA*_ group had a higher average weight than the T_*SA*_ group after one week of study. At the end of the second week, mice in the T_*LB*_-fed group had higher mean weights (20.71 ± 1.28 g) than those in the T_*SA*_ (20.14 ± 1.84 g) and T_*LBSA*_ (19.55 ± 0.55 g) groups. It was also observed that while there was a slight decrease in mean weight for the T_*SA*_ group from the first to the second week of this study (21.65 to 20.14 g), there was a slight increase in mean weight in the T_*LBSA*_ group (19.42 to 19.55 g) within the same period. This suggests that *L. bulgaricus* KLDS 1.0207 could be useful in not only lowering weight loss in study animals within the two-week study period, but its administration could result in weight gain (*P* > 0.05). For the *E. coli* ATCC25922-fed group, the T_*LBEC*_ group had a higher mean weight (14.77 ± 9.86 g) than the T_*EC*_ group (14.45 ± 9.70 g) after two weeks of study, although this was significantly less (*P* < 0.05) than the T_*LB*_ (20.71 ± 1.28 g) and control (21.53 ± 1.24 g) groups (Figure 3 and Supplementary Table S3). In addition, the weight of mice in the T_*LBEC*_ group was more than that of the T_*EC*_ group after one week of study, and these were comparable to mice weights in the T_*LB*_ and control groups.

### Blood Biochemical Analyses

**FIGURE 4:**
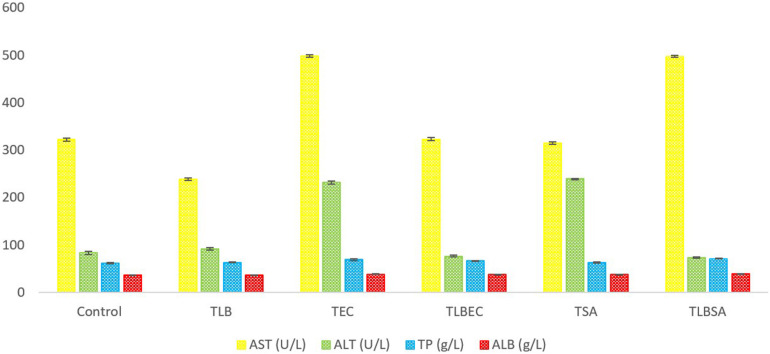
Aspartate aminotransferase (AST), alanine aminotransferase (ALT), total protein (TP), and albumin (ALB) levels (U/L) of BALB/c mice in the control, and all trial groups. All values were obtained in triplicate and expressed as Mean ± SD.

After *E. coli* infection (T_*EC*_), mice AST levels increased significantly from 322 ± 3.3 U/L (control) to 498 ± 2.94 U/L (model group) (*P* < 0.05). A similar increase was observed in ALT levels (from 83 ± 3.65 U/L to 239 ± 1.1.63 U/L, respectively) (Figure 4). However, these parameters were restored to levels similar to the control and T_*LB*_ group when *L. bulgaricus* KLDS 1.0207 was administered alongside *E. coli* ATCC25922 (T_*LBEC*_), demonstrating that *L. bulgaricus* KLDS1.0207 could be effective in preventing *E. coli* ATCC25922 infection activities. Total protein (TP) and albumin (ALB) levels were also elevated after *E. coli* infection compared to the control (*P* > 0.05) but were partially reversed in the T_*LBEC*_ group. After *S. aureus* administration, AST and ALT levels were significantly elevated (*P* < 0.05) compared to the control and the T_*LB*_ groups. These parameters were, however, reversed to normalcy in the T_*LBSA*_ group. Total protein and albumin levels were also returned to normal in the T_*LBSA*_ group. In all, results showed that *L. bulgaricus* KLDS 1.0207 was effective in improving these parameters after administration of *E. coli* and *S. aureus in vivo.*

**FIGURE 5:**
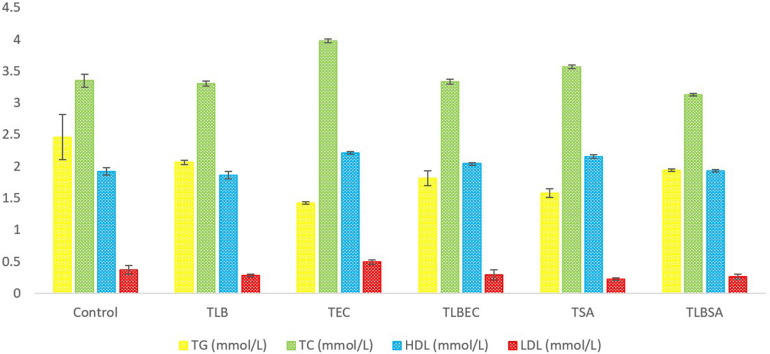
Total glucose (TG), total cholesterol (TC), high-density lipoprotein (HDL), and low-density lipoprotein (LDL) levels (mmol/L) of BALB/c mice in the control and all trial groups. All values were obtained in triplicate and expressed as Mean ± SD.

**FIGURE 6:**
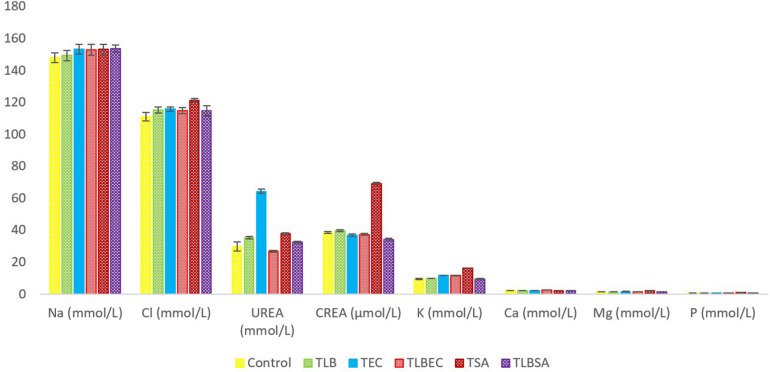
Mineral levels of BALB/c mice in the control and all trial groups. All values were obtained in triplicate and expressed as Mean ± SD.

Mice TG levels in the T_*EC*_ (1.42 ± 0.02 mmol/L) and T_*SA*_ (1.58 ± 0.07 mmol/L) groups were significantly lowered (*P* < 0.05) compared to the control group (2.46 ± 0.36 mmol/L). However, these levels were partially reversed in the T_*LBEC*_ (1.81 ± 0.12 mmol/L) and T_*LBSA*_ (1.94 ± 0.02 mmol/L) groups, suggesting that *E. coli* and *S. aureus* pathogenicity not only lower TG levels in mice but that *L. bulgaricus* KLDS 1.0207 administration could be effective in reversing abnormal TG levels (Figure 5). More so, HDL and LDL levels were significantly altered after *E. coli* and *S. aureus* administration (*P* < 0.05), but they were reversed to levels similar to that of the control group when *L. bulgaricus* KLDS 1.0207 was fed alongside these pathogens (T_*LBEC*_ and T_*LBSA*_). Urea levels increased significantly (*P* < 0.05) after *E. coli* infection (T_*EC*_) compared to the control (64.23 ± 1.34 and 29.63 ± 2.81 mmol/L, respectively). This was reversed in the T_*LBEC*_ group (26.83 ± 0.54 mmol/L). Similar trends were observed in the AST and ALT levels in the model (T_.S.A._ and T_.E.C._) and prevention groups (T_*LBSA*_ and T_*LBEC*_). Creatinine levels in the T_*SA*_ group (69.20 ± 0.54 μmol/L) were also returned to normalcy in the T_*LBSA*_ group (34.18 ± 0.55 μmol/L). Na, K, and Cl levels were also improved in the T_*LB*_ group compared to the control and reversed in the T_*LBEC*_ and T_*LBSA*_ groups compared to the T_*EC*_ and T_*SA*_ model groups, respectively (Figure 6).

## Discussion

### Antimicrobial Activity

As the global population soars, so has the demand for food. In the food and agricultural sectors, the use of chemicals poses health risks and is gradually being discontinued in many industrialized countries. As an alternative, food and agricultural product preservatives from environment-friendly sources like lactic acid bacteria (LAB) are being embraced (Fhoula et al., 2013; Ma et al., 2019). As probiotics, some LABs have improved health and prevented infection activities facilitated by enteropathogenic bacteria (Bian et al., 2015, 2016). However, because of strain-specific effects, only selected LAB candidates obtained from initial screening protocols are further studied (Yan et al., 2020). The current study evaluated the antimicrobial effects of the CFS of *L. bulgaricus* KLDS 1.0207 against two foodborne pathogens after subjection to enzymatic actions and pH changes. It was demonstrated that the CFS inhibited the proliferation of both pathogens, and this was not significantly affected by enzymatic activities (*P* > 0.05). As expected, the antimicrobial effects decreased as pH tended toward neutrality. Antimicrobial substances produced by LABs is a known mechanism for exerting probiotic effects against harmful microbes in the intestinal tract and thus have immense preservative, medical and biotechnological properties (Okereke et al., 2012). Earlier, Bian et al. (2015) showed that antimicrobial substances produced by *L. helveticus* KLDS 1.8701 could inhibit a range of foodborne pathogens, especially *L. monocytogenes* ATCC119115. Also, *L. bulgaricus* KLDS 1.0207 can produce sufficient pathogen-inhibiting organic acid (lactic and acetic acids) *in vitro*, and this increased with carbon source supplementation (Evivie et al., 2019). Our recent report and findings from the current study strongly suggest that the inhibitory properties exhibited by this strain can be attributed to acid metabolites. This further confirms that this strain can be a potential source of bio-preservative in the food and allied industries. Further characterization of the antimicrobial substances produced, like bacteriocins and their specific mechanisms of action, is recommended for future research, as this data will support the use of *L. bulgaricus* KLDS 1.0207 as a potential functional food ingredient. In addition, the use of ‘omics’ techniques may give further insights into the antimicrobial pathways of *L. bulgaricus* KLDS 1.0207 (Evivie et al., 2020). Besides, preliminary trials showed that *L. bulgaricus* KLDS 1.0207 could inhibit the growth of the vaginal pathogen, *Gardnerella vaginalis* ATCC14018 (Supplementary Table S1), reputed as a primary causal organism in the etiology of bacterial vaginosis (BV) (Peebles et al., 2019). Its CFS also had antimicrobial effects after pH alterations and enzymatic actions. Although the exact mechanism by which this vaginal pathogen was inhibited is not known, the current study can only hypothesize at this point that *L. bulgaricus* KLDS 1.0207 may have potential use in the medical and pharmaceutical industries as a BV-inhibiting agent.

### Tolerance Assay

In the present study, the tolerance levels of *L. bulgaricus* KLDS 1.0207 in acid and bile salts were assessed to evaluate its possible probiotic effects. Our results show that this strain can survive through harsh stomach environment in adequate amounts (log 6.96 ± 0.02 CFU mL^–1^ after three hours) to have specific functions. The ability of sufficient quantities of LAB strains to transit the unfavorable acidic conditions of the stomach to the distal end of the ileum where it can proliferate to have beneficial effects is an essential indication of probiotic efficacy (Ljungh and Wadström, 2006; Rupa and Mine, 2012). This has been demonstrated in many previous studies involving *Lactobacillus, Bifidobacterium, and Streptococcus* strains (Thantsha et al., 2012; Ashraf and Smith, 2016; Evivie et al., 2017; Tang and Zhao, 2019). A recent study assessing the biological and antidiabetic properties of *Lactobacillus* strains (including *L. bulgaricus* KLDS 1.0207) demonstrated that their acid and bile tolerant properties have the potentials for adhesion to or colonization of host intestine, which could endow them with anti-diabetic properties (Yan et al., 2020). Furthermore, in evaluating the tolerance levels of several promising commercial strains, Ashraf and Smith (2016) recently showed that some *Lactobacillus bulgaricus* strains could survive effectively under acid and bile conditions for up to 12 h, which again demonstrates the efficacy of specific strains to function as potential probiotics. *In vivo* studies are also required to validate these findings further.

### Cytotoxic Activities Against RAW264.7 Cells

Imbalances in the gut microbiota have been implicated in the onset and progression of inflammatory bowel disease (IBD) (Ryan et al., 2020). Accumulating evidence recommended the use of probiotics in IBD therapy against the traditional use of antibiotics (Liu M. et al., 2019). Also, Choi et al. (2019) recently posited that lipopolysaccharides (LPS) are known to worsen the IBD status of sufferers. From the preceding, research attention has been concerted toward the possible use of probiotic strains in LPS activities, thus alleviating IBD in humans. In this study, the LPS-lowering effects of *L. bulgaricus* KLDS 1.0207 were investigated, given its further application as a potential therapeutic ingredient. Results show that an increase in *L. bulgaricus* KLDS 1.0207 cell count resulted in a significant decrease in RAW264.7 inflammatory activities. Previously, *L. bulgaricus* KLDS 1.0207 has been shown to effectively alleviate lead (Pb) toxicity *in vitro* and *in vivo*, thus supporting the hypothesis that this strain could have promising anti-inflammatory uses in the food and medical industries (Li et al., 2017). It should be noted that the specific mechanisms by which this effect was exerted is unknown and warrants further studies.

### Weight of Animals

In the present study, the administration of *L. bulgaricus* KLDS 1.0207 prevented significant weight loss (compared to the model disease groups) and, at other times, resulted in weight gain (compared to the control). This may be due to one or a combination of the *in vitro* inhibitory activities earlier reported in this study – CFS antimicrobial activities, organic acid production, survivability under acid, and bile salt conditions. Similar trends were observed in the activities of *L. bulgaricus* 151 in DSS-induced mice trials (Wasilewska et al., 2019). Herein, the researchers also interestingly observed a slight limited loss in weight, which is in agreement with what is reported at present. Previously, the weight of studied animals was shown to improve after probiotic administration, thus suggesting that specific single or multiple-dose strains could suppress pathogenic processes and pathways that promote weight loss (Aboderin and Oyetayo, 2006; Niamah et al., 2017). In a preliminary trial, it was demonstrated that a co-culture of *S. thermophilus* KLDS 3.1003 and *L. bulgaricus* KLDS 1.0207 had better effects on the weight of study animals than when both strains were administered separately (data not shown). Based on our previous and current findings, we propose that this co-culture could be used in formulating dairy products that have desirable antagonistic effects against selected foodborne pathogens. Further studies to investigate this hypothesis are thus recommended. These findings also suggest that *L. bulgaricus* KLDS 1.0207 administration was more effective toward *S. aureus* ATCC25923 inhibition than *E. coli* ATCC25922.

### Biochemical Analyses

Hypercholesterolemia is a known leading cause of coronary heart diseases (CHD), with the World Health Organization (WHO) predicting that it would remain a major cause of death worldwide until 2030 (Thomas and Rich, 2007; Mohania et al., 2013; Dehkohneh et al., 2019). The possibility of experiencing a cardiac arrest is three times higher in hypercholesterolemic individuals than those with normal blood lipid levels (Kumar et al., 2012). Although the ameliorative effects of *L. bulgaricus* KLDS 1.0207 against Pb-toxicity had been earlier reported, the present study is the first to report its preventive effects against *E. coli* and *S. aureus* pathogens by assessing *in vivo* blood biochemical parameters. It was revealed that the total cholesterol (TC) levels in the T_*SA*_ and T_*EC*_ groups were significantly higher compared to the control group (*P* < 0.05). These levels were reversed in the T_*LBSA*_ and T_*LBEC*_ prevention groups to those similar to the control group, thus demonstrating that *L. bulgaricus* KLDS 1.0207 could have cholesterol-lowering effects. Raised cholesterol levels account for an estimated 2.6 million deaths globally, and a 1% reduction in TC levels results in a 2.3% reduction in coronary related risks (Baroutkoub et al., 2010; World Health Organization [WHO], 2018). Recently, tropical fruit-derived *L. plantarum* strains lowered both blood glucose and total cholesterol levels in Winstar rats after 14 days of administration (*P* < 0.05), suggesting that probiotics can improve host health status by modulating biochemical parameters (da Costa et al., 2019). Also, the AST and ALT parameters are indicators of the proper functioning of vital organs like the liver (Hezaveh et al., 2019; Mirmozaffari, 2019). We show that although oral administration of these food pathogens negatively altered these parameters (T_*SA*_ and T_*EC*_ groups), the consumption of *L. bulgaricus* KLDS 1.0207 (T_*LBSA*_ and T_*LBEC*_ groups) ameliorated this situation. Although this is a welcome development, further studies are required to confirm our findings.

Also, HDL and LDL levels in the T_*SA*_ and T_*EC*_ groups were significantly altered compared to the control group, signaling the pathogenicity of both organisms. However, these anomalies were reversed when *L. bulgaricus* KLDS 1.0207 was administered with the pathogens (T_*LBSA*_ and T_*LBEC*_ groups). LDL has also been implicated in incidences of cardiovascular diseases (Mattiuzzi et al., 2020). These findings align with a recent preliminary study showing that *L. paracasei* increased and decreased HDL and LDL levels in seven hypercholesterolemic patients (Chaiyasut et al., 2019). It is interesting to note that no high-fat diets (HFD) were administered in the present study before significant anomalies in the HDL and LDL levels were observed (*P* < 0.05). Recently, Aziza et al. (2019) assessed serum biochemical parameters in mice without feeding HFD and observed marked changes between the model (*E. coli*) and test groups. This again strengthens our position that food pathogens can disrupt many biochemical parameters (including HDL and LDL), and HFD does not need to be administered to observe significant variations in HDL, LDL, TG, and others.

Similarly, micronutrient levels that were disrupted by pathogen administration were at least partially reversed by *L. bulgaricus* KLDS 1.0207 administration alongside the pathogens as a prevention protocol. In the future, the mechanisms behind these exerted effects will require further investigations to have a clear understanding of how this strain improves biochemical parameters. Also, the *in vivo* antimicrobial effect of this strain can be explored by considering more indexes related to toxin-producing or gut barrier integrity with more doses of the strain, and inclusion of positive and negative control strains. It may also be important to assess modulations in the gut microbiota of mice in these respective groups in the future to understand the mechanisms by which *L. bulgaricus* KLDS 1.0207 restores normalcy to levels similar to the control and T_*LB*_ groups. In all, findings from this study present *L. bulgaricus* KLDS 1.0207 as a tolerant strain with potential antimicrobial, cytotoxic, lipid-regulating, and anti-hypercholesterolemic properties which could endow it with promising future applications.

## Conclusion

Research investigating the effects of potential LAB strains against notable foodborne pathogens are still ongoing. This study assessed the *in vitro* antimicrobial, cytotoxic, and tolerance properties of *L. bulgaricus* KLDS 1.0207. Besides, the *in vivo* ameliorative effects of this strain on weight and serum biochemical parameters were evaluated. Results showed that its CFS had high antimicrobial effects against both pathogens even after enzymatic actions and pH alterations. It was also tolerant in simulated acid and bile salts. Finally, this LAB strain showed strong cytotoxic effects in murine RAW264.7 cells *in vitro* and improved weight and blood biochemical parameters *in vivo*. These findings put together, support the hypothesis that the *L. bulgaricus* KLDS 1.0207 is a probiotic strain with potential applications in the food and allied industries. Further targeted *in vivo* studies elucidating the mechanisms behind these effects as well as how these activities improve gut barrier integrity are warranted.

## Data Availability Statement

All datasets presented in this study are included in the article/Supplementary Material.

## Ethics Statement

The animal study was reviewed and approved by the NEAU Animal Ethics Committee (SRM-06).

## Author Contributions

GH and SE conceived the study and acquired the funding for this project. SE, SL, AA, FL, and BL developed the method protocols. GH and FL validated the protocols. SE carried out formal analyses and wrote the original draft manuscript. SE and AA carried out the animal experiments. SE, FL, BL, and AA managed the resources for this study. SE, AA, and BL collected all data and administered it. SE, GH, and BL revised and edited the original draft. GH supervised the project. All authors have read and agreed to the published version of the manuscript.

## Conflict of Interest

The authors declare that the research was conducted in the absence of any commercial or financial relationships that could be construed as a potential conflict of interest.
