# Supplementary material for: Lactobacillus delbrueckii subsp. bulgaricus KLDS 1.0207 Exerts Antimicrobial and Cytotoxic Effects in vitro and Improves Blood Biochemical Parameters in vivo Against Notable Foodborne Pathogens
Source: Front Microbiol. 2020 Sep 24;11:583070. doi: 10.3389/fmicb.2020.583070 (PMC7541842; doi:10.3389/fmicb.2020.583070)
Supplement: Supplementary file 1 [file Table_1.DOCX]

| Remaining antimicrobial activity (%) | | |
| --- | --- | --- |
| Treatment | Control | *G. vaginalis* ATCC14018 |
| Catalase | 100±0^a^ | 93.11±0.33^a^ |
| Proteinase K | 100±0^a^ | 100±0^a^ |
| Papain | 100±0^a^ | 100±0^a^ |
| pH 3.5 | 100±0^a^ | 100±0^a^ |
| pH 4.0 | 100±0^a^ | 100±0^a^ |
| pH 5.0 | 100±0^a^ | 70.37±0.48^b^ |
| pH 6.0 | 100±0^a^ | 8.84±0.20^c^ |
| pH 6.5 | 100±0^a^ | 0±0^d^ |
| pH 7.0 | 100±0^a^ | 0±0^a^ |

Values were expressed as Mean ± SD

All values were determined in triplicate

Values with the same alphabet along same row are not significantly different (*P*>0.05)

Supplementary Table S1: Effects of enzymatic actions and pH changes on the antimicrobial activity of the CFS of *L. bulgaricus* KLDS 1.0207 against *G. vaginalis* ATCC14018. No antimicrobial activities were observed at pHs 6.5 and 7.0
