# Supplementary material for: Lactobacillus delbrueckii subsp. bulgaricus KLDS 1.0207 Exerts Antimicrobial and Cytotoxic Effects in vitro and Improves Blood Biochemical Parameters in vivo Against Notable Foodborne Pathogens
Source: Front Microbiol. 2020 Sep 24;11:583070. doi: 10.3389/fmicb.2020.583070 (PMC7541842; doi:10.3389/fmicb.2020.583070)
Supplement: Supplementary file 2 [file Table_2.DOCX]

| Period | Control | Control | Control | Control |
| --- | --- | --- | --- | --- |
| Week 0 | 22.00^b^ | 20.84^e^ | 19.34^e^ | 21.42^b^ |
|  | 20.90^d^ | 23.84^b^ | 20.19^d^ | 21.00^b^ |
|  | 23.92^a^ | 21.63^e^ | 20.56^c^ | 18.18^f^ |
|  | 20.89^d^ | 22.61^c^ | 20.00^d^ | 22.68^a^ |
| Average | 22.43 | 22.23 | 20.02 | 20.82 |
| SD | 1.29 | 1.29 | 0.51 | 1.90 |
|  | Control | T_LB_ | T_SA_ | T_LBSA_ |
| Week 1 | 24.92^a^ | 23.79^b^ | 21.19^c^ | 20.45^c^ |
|  | 23.34^a^ | 22.00^d^ | 20.26^d^ | 19.38^d^ |
|  | 21.98^b^ | 24.89^a^ | 20.85^c^ | 19.83^d^ |
|  | 22.60^b^ | 24.50^a^ | 24.28^a^ | 18.00^f^ |
| Average | 23.21 | 23.78 | 21.65 | 19.42 |
| SD | 1.27 | 1.28 | 1.80 | 1.04 |
| Week 2 | 23.25^a^ | 21.43^e^ | 19.94^d^ | 19.42^d^ |
|  | 21.32^c^ | 19.04^g^ | 18.46^f^ | 19.36^d^ |
|  | 20.29^d^ | 21.95^d^ | 19.40^e^ | 20.34^c^ |
|  | 21.27^c^ | 20.43^e^ | 22.74^b^ | 19.07^e^ |
| Average | 21.53 | 20.71 | 20.14 | 19.55 |
| SD | 1.24 | 1.28 | 1.84 | 0.55 |

Values with the same alphabet along the same column are not significantly different (P>0.05)

Supplementary Table S2: Weekly weights of study animals before and after *S. aureus* ATCC25923 infection. Despite this pathogen’s deleterious effects, *L. bulgaricus* KLDS 1.0207 administration (T_LBSA_) inhibited weight loss to levels comparable with the C, T_LB_, T_SA_ groups (*P* > 0.05).
