# Supplementary material for: Lactobacillus delbrueckii subsp. bulgaricus KLDS 1.0207 Exerts Antimicrobial and Cytotoxic Effects in vitro and Improves Blood Biochemical Parameters in vivo Against Notable Foodborne Pathogens
Source: Front Microbiol. 2020 Sep 24;11:583070. doi: 10.3389/fmicb.2020.583070 (PMC7541842; doi:10.3389/fmicb.2020.583070)
Supplement: Supplementary file 3 [file Table_3.DOCX]

| Period | Control | Control | Control | Control |
| --- | --- | --- | --- | --- |
| Week 0 | 22.00^b^ | 20.84^e^ | 21.41^b^ | 25.00^a^ |
|  | 20.90^d^ | 23.84^b^ | 22.96^a^ | 22.04^c^ |
|  | 23.92^a^ | 21.63^e^ | 21.12^b^ | 20.89^d^ |
|  | 20.89^d^ | 22.61^c^ | 21.77^b^ | 23.08^b^ |
| Average | 22.43 | 22.23 | 21.82 | 22.75 |
| SD | 1.29 | 1.29 | 0.81 | 1.75 |
|  | Control | T_LB_ | T_EC_ | T_LBEC_ |
| Week 1 | 24.92^a^ | 23.79^b^ | 20.51^c^ | 24.12^a^ |
|  | 23.34^a^ | 22.00^d^ | 21.54^b^ | 22.82^c^ |
|  | 21.98^b^ | 24.89^a^ | 21.24^b^ | 22.05^c^ |
|  | 22.60^b^ | 24.50^a^ | 22.49^a^ | 22.46^c^ |
| Average | 23.21 | 23.78 | 21.45 | 22.86 |
| SD | 1.27 | 1.28 | 0.82 | 0.90 |
| Week 2 | 23.25^a^ | 21.43^e^ | 18.59^d^ | 19.09^e^ |
|  | 21.32^c^ | 19.04^g^ | 18.36^d^ | 19.43^e^ |
|  | 20.29^d^ | 21.95^d^ | 20.85^c^ | 20.56^d^ |
|  | 21.27^c^ | 20.43^e^ | - | -- |
| Average | 21.53 | 20.71 | 14.45 | 14.77 |
| SD | 1.24 | 1.28 | 9.70 | 9.86 |

Values with the same alphabet along the same column are not significantly different (P>0.05)

Supplementary Table S3: Weekly weights (g) of study animals before and after *E. coli* ATCC25922 infection. After two weeks of study, slight weight gain was recorded in the T_LBEC_ group compared to the model group (T_EC_) (*P* > 0.05)
